# Managing tensions in assessment: moving beyond either–or thinking

**DOI:** 10.1111/medu.13656

**Published:** 2018-10-05

**Authors:** Marjan J B Govaerts, Cees P M van der Vleuten, Eric S Holmboe

**Affiliations:** ^1^ Department of Educational Development and Research Faculty of Health, Medicine and Life Sciences Maastricht University Maastricht the Netherlands; ^2^ Accreditation Council for Graduate Medical Education Chicago Illinois USA

## Abstract

**Context:**

In health professions education, assessment systems are bound to be rife with tensions as they must fulfil formative and summative assessment purposes, be efficient and effective, and meet the needs of learners and education institutes, as well as those of patients and health care organisations. The way we respond to these tensions determines the fate of assessment practices and reform. In this study, we argue that traditional ‘fix‐the‐problem’ approaches (i.e. either–or solutions) are generally inadequate and that we need alternative strategies to help us further understand, accept and actually engage with the multiple recurring tensions in assessment programmes.

**Methods:**

Drawing from research in organisation science and health care, we outline how the Polarity Thinking™ model and its ‘both–and’ approach offer ways to systematically leverage assessment tensions as opportunities to drive improvement, rather than as intractable problems. In reviewing the assessment literature, we highlight and discuss exemplars of specific assessment polarities and tensions in educational settings. Using key concepts and principles of the Polarity Thinking™ model, and two examples of common tensions in assessment design, we describe how the model can be applied in a stepwise approach to the management of key polarities in assessment.

**Discussion:**

Assessment polarities and tensions are likely to surface with the continued rise of complexity and change in education and health care organisations. With increasing pressures of accountability in times of stretched resources, assessment tensions and dilemmas will become more pronounced. We propose to add to our repertoire of strategies for managing key dilemmas in education and assessment design through the adoption of the polarity framework. Its ‘both–and’ approach may advance our efforts to transform assessment systems to meet complex 21st century education, health and health care needs.

## Abstract 2

The authors argue that traditional ‘either‐or’ solutions to assessment problems need to be replaced with ‘both‐and’ strategies if we are to advance our efforts to transform assessment in the era of competency‐based education.

## Introduction

Ultimately, the purpose of health professions education (HPE) is to benefit the quality of health systems by transforming learners into qualified professionals who not only have achieved standards of competence that are acceptable to the profession and the community, but are first and foremost committed to excellence, lifelong learning and the ongoing advancement of the field and high‐quality care.^1^, ^2^ In HPE, as in any other education system, assessment is seen as fundamental to achieving these education goals.^3^ In order to meet the demands of rapidly changing health care systems and the expectations of the public, approaches to education and assessment have evolved radically over the past few decades. Recent approaches to assessment reform aim to align assessment practices with models of outcome‐based or competency‐based education (CBE).^4^, ^5^, ^6^ Within the framework of CBE, assessment typically focuses on fostering the development of professional competence and ensuring robust decision making about learners’ and physicians’ fitness for practice. Competence‐based assessment systems are typically complex as they rely on programmes of assessment that include multiple methods (standardised as well as non‐standardised), contexts and assessors, and must be embedded in highly complex education as well as health care systems.^6^, ^7^ Assessment systems are thus bound to be rife with tensions as they must fulfil formative and summative assessment purposes, be efficient and effective, and meet the needs of learners, education institutes, patients and health care organisations. Addressing these competing demands requires systems that are standardised as well as authentic, that allow for control as well as trust, and that foster cultures that enable and value learning as well as high‐quality performance. As increasing pressures to reform HPE accentuate these multiple polarities inherent in modern assessment programmes, the resulting tensions seem to become even more salient in times of scarcity (increasing demands for high‐quality performance while reducing costs) and plurality (e.g. multiple perspectives on the ‘what’ and ‘how’ in education reform), as illustrated by ongoing debates about what is right about how to educate and assess health professionals.^8^, ^9^ Scholarly papers criticising or favouring concepts of CBE and workplace‐based assessment, and discussions about the role of subjectivity and qualitative assessment approaches may very often reflect competing yet coexisting goals, tasks and roles, as well as individuals’ emotions and cognitive frames shaped by cultural and contextual factors.^10^, ^11^, ^12^, ^13^ The way we respond to these tensions may very well be a fundamental determinant of the fate of assessment practices and reform.

A common response to tensions in organisational systems is the application of the so‐called ‘contingency approach’ whereby researchers seek to capture a multifaceted reality with a perfect, internally consistent theory, and practitioners look upon tensions as ‘problems that need [to] and can be fixed’.^14^, ^15^ Through the contingency lens, challenging tensions in assessment systems become problems that will be solved and disappear if we can agree upon the single right answer. We then typically start searching for ‘if–then’ insights, striving to identify under which conditions either A or B needs to be emphasised or selected (e.g. standardisation or authenticity; quantitative or qualitative assessment approaches). This approach typically results in ‘either–or’ discussions, enabling rational decision making about assessment design and implementation. For example, using findings from assessment research as well as careful analysis of the US medical education context, Hanson and colleagues wrote a powerful plea for replacing numbers (grades) with words (narratives) to enable the achievement of education and assessment goals.^16^ Similarly, assessment experts argue that different information management strategies are to be used depending on assessment purposes and rationale,^6^ that we should refrain from grading if we use assessment for formative purposes,^17^, ^18^, ^19^, ^20^ that we should focus on standardised assessments and criteria‐based grading in the assessment of learning in order to ensure that our graduates are equivalent and fit for practice,^21^, ^22^, ^23^, ^24^, ^25^ and that we should favour qualitative over quantitative assessment approaches if we want to capture professional competence.^26^ Proposed solutions then typically include measures to ‘overcome’ barriers to the successful implementation of the assessment ideal or to provide arguments to illustrate the fallibility of others’ views and thinking.

It is increasingly recognised, however, that traditional ‘fix‐the‐problem’ approaches to coping with multiple tensions are generally inadequate as they fail to *sustainably* address the quantity and complexity of polarities in organisations. Likewise, the widespread ongoing and recurring debates in the HPE community suggest that many assessment tensions do not represent problems that can be resolved, but polarities that need to be carefully managed. This is the core premise of the Polarity Thinking™ (Polarity Partnerships, LLC, Auburn, CA, USA) approach. This approach may offer promising and alternative ways of responding to tensions in complex organisational settings and help us further understand, accept and actually engage with the enduring tensions we face in education and assessment systems.

The purpose of this paper was to outline how Polarity Thinking™ may help assessment reform move forward by systematically leveraging assessment polarities as opportunities to drive improvement rather than as intractable problems. Drawing from literature in organisation science^14^, ^15^, ^27^, ^28^ and health care reform,^29^, ^30^ we will present a brief overview of the key concepts in Polarity Thinking™, followed by the identification and categorisation of common tensions in assessment. We will finally describe how the model can be used to map and manage key polarities in assessment.

## Reframing problems as polarities

The foundational premise of Polarity Thinking™ is the view that tensions must be accepted in order to achieve long‐term success and sustained transformation because they are inherent to human behaviours in complex, dynamic and ambiguous systems.^15^, ^27^, ^28^, ^29^, ^30^ Key principles underpinning this perspective are summarised in Box 1.

Box 1 **Adapted from Johnson,^28^ and Wesorick and Shaha^30^**

**Basic polarity principles**

1. Polarities are interdependent pairs of different values or points of view (so‐called ‘poles’)
2. The different values or poles need each other over time to reach the higher goal neither can achieve alone, even though there is tension between them
3. Both poles bring positive outcomes or an ‘upside*’*
4. Both poles have a potential ‘downside*’*
5. If one pole is neglected, there will be negative outcomes
6. Polarities are intrinsic to complex systems and organisations. Polarities are not problems to be solved; tensions between poles are unavoidable and must be leveraged

Basically, polarities are two (or more) values or alternative views (called ‘poles’) that may appear as opposite or competitive but are interdependent and need each other to achieve a goal neither can reach alone.^28^ Each of the poles brings positive outcomes to the overarching goals to be achieved, whereas emphasising one pole at the expense of the other will result in negative outcomes. In organisational systems, the coexistence of these different yet interrelated poles is commonly experienced as ‘tension’. Polarity Thinking™ entails a ‘both–and’ mindset rather than ‘if–then’ thinking or ‘either–or’ solutions in managing these tensions or dilemmas. Within assessment systems, assessment for learning (formative) and assessment of learning (summative) are typical examples of polarities, creating tensions between subsystems with different (sub)goals, functions and expectations. We need both, however, to achieve our ultimate goals of ensuring robust judgements and high‐quality learning for high‐quality patient care. In assessment, there can be no formative assessment without summative assessment: formative assessment is always about identifying the gap between actual performance and expected performance or required standards, defined as summative assessment criteria. Summative assessment is thus always embedded in formative assessment, either implicitly or explicitly.^31^ Likewise, people will argue that valid, fair assessment of learning is not possible without ongoing formative assessment.

Reframing problems as polarities (or paradoxes) is fairly well established in organisation science and research shows that leaders and organisations that manage organisational tensions using this perspective outperform those that do not.^32^, ^33^, ^34^ More recently, Polarity Thinking™ models have been successfully introduced to optimise health care reform, as traditional change efforts – aiming at fixing a polarity as a problem to be solved – did not result in the sustainable achievement of desired outcomes.^30^, ^35^ Likewise, we propose the Polarity Thinking™ perspective may enable us to face and manage increasingly complex conditions for the assessment reform that is necessary to meet the rapidly changing demands of learners, organisations and the public.

## Polarities and tensions in assessment: categorisation and debates

**Figure 1:**
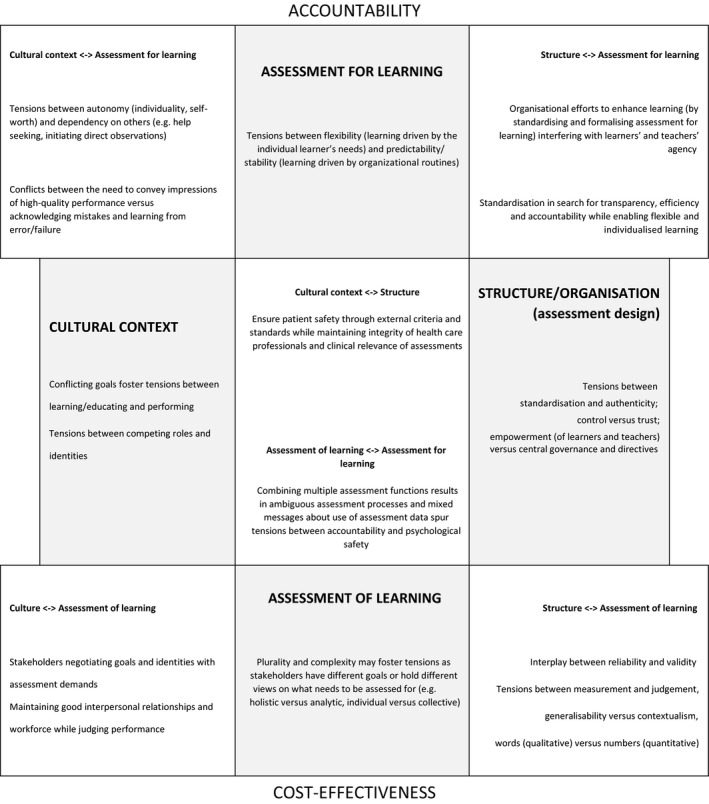
Categorisation of common assessment tensions (after Lewis and Smith^15^). Exemplars of key tensions that arise within and between core elements in assessment systems, driven by increasing pressures for accountability and cost‐effectiveness. Shaded boxes present potential tensions within core elements, such as tensions within assessment for learning and assessment of learning, the structure and organisation of assessment (assessment design) and the cultural context in which assessment is embedded. The central and corner boxes present exemplars of tensions arising between these core elements

Based on our collective experience with assessment and drawing from the assessment literature within and outside the domain of HPE, we identified and categorised common polarities and tensions in assessment systems (Fig. 1). It is not our intent to provide a systematic review of assessment research; rather, the purpose of our paper is to highlight exemplars of specific assessment polarities and tensions in order to demonstrate how Polarity Thinking™ can assist in addressing current tensions in assessment (in relation to CBE in particular).

We defined core activities and elements in assessment systems (i.e. assessment *for* learning; assessment *of* learning; assessment structure and organisation; cultural context) and identified tensions that arise within and between these elements. In fact, many tensions in assessment systems seem to reflect the complex interrelationships and interactions among these core elements, not infrequently driven by increasing pressures for accountability and cost‐effectiveness.

For many decades, the significance of formative assessment as a powerful driver for learning has been acknowledged.^17^, ^36^, ^37^, ^38^ With its emphasis on feedback, learning processes and student engagement in assessment, it aims to develop students and trainees into efficient, lifelong learners. High‐quality formative assessments direct attention to goals to be achieved and how to achieve them, and thus act as incentives to align study and practice with the needs of individual learners. *Assessment for learning* tensions may then become salient if the emergence of individual learning trajectories involves flexible curricula and the reorganisation of work processes, impacting not only on the (scarce) financial, material and administrative resources of schools and health care institutions, but also on the quality of patient care.^39^, ^40^, ^41^ The need to create flexible and dynamic learning while maintaining stable working routines in the interwoven systems of education and health care and competing goals of efficiency and effectiveness may then create conflict (‘strain’) and ambiguity regarding assessment strategies.

*Assessment of learning* has for long been the almost exclusive focus of assessment content and approaches; issues of accountability, fairness and equivalence in the context of summative assessment have dominated assessment development and research in HPE until very recently. Tensions within *assessment of learning* may stem from the plurality of stakeholders’ views on how to define what needs to be assessed in order to ensure high‐quality care. Scholars, as well as practitioners, may hold different views on what constitutes ‘professional competence’^42^, ^43^ and on how conceptualisations of competence are to be translated into assessment requirements.^44^ The complexity of patient care may drive tensions between the individual and the collective because individual competence needs to be assured although teamwork and organisation performance largely contribute to patient outcomes.^45^, ^46^ Similarly, efforts to reform assessment in CBE may drive tensions between cost‐effectiveness (focus on ‘fitness for practice’ – being ‘good enough’) and excellence^47^, ^48^ and/or between holistic and analytic approaches to competence. Although it is widely acknowledged, for example, that *‘*professional competence is more than a demonstration of isolated competencies*’* and that professional competence is to be defined as the inseparable and integrated use of knowledge, skills, norms, values, judgement and reasoning to serve the health care needs of the community,^49^ the development of professional competence may require the description and evaluation of separate competencies or competency domains to ensure intended outcomes for high‐quality care.^5^, ^6^, ^50^, ^51^

Tensions when designing (*structuring and organising*) assessment will surface if the achievement of the desired outcome requires different competing, yet coexisting processes and systems. Common tensions operate between standardisation and authenticity, or between (external) control (e.g. regulatory bodies) and trust in local assessment expertise as a critical resource in assessment decisions. Competing demands of internal and external stakeholders may raise tensions between equivalency (such as through the implementation of national licensing tests) and alignment with the medical school's or workplace curriculum. Serious dilemmas may then arise in contexts in which efforts to ensure quality of assessment for accountability through top‐down, prescriptive (national) mandates or tests are interpreted to signify loss of programme autonomy and professional integrity, whereas non‐compliance may result in the loss of programme accreditation.^52^, ^53^

Obviously, well‐described and well‐researched tensions between assessment design and assessment *for* and *of* learning reflect competing demands of reliability, validity, feasibility and acceptance. These tensions surface in debates around qualitative versus quantitative assessment approaches,^26^ the role of ‘objectification’ (objectivity) versus subjectivity^54^, ^55^ and a psychometric versus ‘edumetric’ or educational approach to assessment design.^56^ At a more micro level, stakeholders’ struggles with grades and narrative assessments, or the use of global rating scales and detailed checklists, illustrate efforts to navigate these tensions within specific assessment contexts. The requirements of accountability and cost‐effectiveness in particular may push towards objectified quantitative assessment outcomes, favouring grades and standardised assessments. As a consequence, tensions may surface through differing, ambiguous and sometimes conflicting assessment strategies such as the ‘standardisation’ of inherently unstandardised assessments (e.g. standardised narratives for authentic assessments),^57^, ^58^ ‘measurement’ of performance‐in‐context through the use of detailed rating scales and task‐specific checklists,^59^, ^60^ or the ‘objectification’ of competence and competence development through the use of entrustable professional activities and milestones.^61^, ^62^

In addition, education accountability may entail a ‘timekeeping function’ of assessment as assessment may (implicitly or explicitly) serve to increase the rate of learning: frequent assessments become ‘stopwatches’, controlling individual progress and the efficiency of learning processes.^63^ Tensions between control and trust, or between standardisation and authenticity may then not only interfere with processes of teaching and learning in classroom settings or work environments,^64^, ^65^, ^66^, ^67^, ^68^ but also impact on stakeholders’ sense of autonomy, agency and engagement with assessment as a tool for learning.^69^ Tensions between assessment design and assessment for learning may equally reflect conflicts between the need to enable individualised learning on the basis of meaningful feedback and the desire to structure and standardise in search of efficiency and accountability. Discussions about the use of numbers (grades) versus words (narratives) may be illustrative in this respect. In general, grades are considered to be the poorest form of feedback, whereas high‐quality feedback is believed to be conveyed through the use of words. However, although the role of quality narratives in feedback for performance improvement is undisputed, research findings show that the standardisation of assessment and provision of grades that reflect progress towards competence may enhance learners’ sense of self‐efficacy and competence, as well as perceptions of fairness and equivalence with respect to levels of competence upon graduation.^20^, ^24^, ^70^, ^71^ Numbers can be very efficiently aggregated into individualised profiles of learners and learners’ progress, supporting self‐assessment and inciting reflection and action for change.^72^ As Tekian et al. state: ‘Under appropriate circumstances, numbers can be more meaningful than a thousand pictures.’^72^ Various stakeholders may thus perceive the provision of numerical assessment data to be essential in maintaining a culture of excellence: numerical data enable the efficient and psychometrically sound ranking of learners, allowing learners to ‘objectively’ demonstrate excellence in a highly competitive environment and allowing stakeholders to defensibly select the ‘best‐performing’ individuals for later admissions decisions. Decisions made when dealing with these tensions are thus inextricably linked with key characteristics of the cultural contexts in which assessment systems are embedded.

Clearly, health care and health care systems represent complex contexts, in which different organisational identities, goals, norms and values may drive tensions between learning and performance. Obviously, the tensions of competing goals in the delivery of high‐quality, efficient patient care, as well as high‐quality, efficient teaching and learning, are likely to become more salient under conditions of limited resources and increasing pressures for accountability.^73^ In health care settings, learning–performance tensions may surface if an individual is required to fulfil the multiple roles of learner as well as health care provider (trainees), or health care provider, coach and assessor (clinicians). In workplace settings, trainees learn through and for work, and assessment of their learning inevitably involves judgement of the quality of their work. Critical feedback can thus feel uncomfortable to learners.^74^ Similarly, assessors’ use of linguistic strategies, such as hedging, in attempts to maintain smooth social interactions and working relationships with their trainees while providing constructive feedback may reflect supervisors’ competing goals and conflicts between internal (towards learners) and external (towards institutions and the public) accountability.^75^, ^76^ At the level of the education institution, the competing goals and demands of different stakeholders may result in similar conflicts between the actual use of assessment results and the assessment purposes communicated to teachers and learners. For instance, although management may support the role of teachers as the profession's gatekeepers, it may at the same time tell staff that there is concern about the retention of students and the related funding of the institution or training programme.^77^ In addition, low or below average ratings are considered unacceptable in some organisations (including in HPE and health care settings) even if they are accurate, and implicit organisational norms and pressures for conformity may be a significant factor in inflating ratings in clinical settings.^78^, ^79^

Interactions between the cultural context and assessment may also spur conflicts between autonomy and independence – core values in the current culture of medicine – and a learning culture that fundamentally values routine direct observation and feedback seeking.^73^, ^74^, ^80^ Tensions between performance (looking good) and learning (being willing to show weaknesses, to admit and learn from failure) may additionally result in learners grappling with the need to engage in trustful relationships, interdependence and collaboration to foster development and expertise, while being competitive and seeking to outperform others in the battle for placements in residency training programmes or top‐tier hospitals and health care organisations.^81^, ^82^

Similar types of tension may surface when integrating assessment for and of learning in assessment programmes. In programmatic assessment approaches, for instance, enhancing developmental assessment functions through frequent low‐stakes performance evaluation and feedback conflicts with the summative use of these assessment data to ensure robust decision making. Learners, as well as teachers, may then face tensions between psychological safety and accountability when experiencing mixed messages, conflicting goals and ambiguous processes. As a result, learners and teachers may perceive low‐stakes assessments as high stakes, which impacts negatively on learning and learning strategies.^69^, ^83^, ^84^, ^85^ Similar tensions may occur at organisational levels: while the enhancement of efficient, self‐regulated lifelong learning is a pressing priority in education reform, accountability pressures may result in education institutions focusing on assessment for the purposes of selection, discipline and control.

Although by no means complete, this overview identifies many conflicting yet interrelated elements across a range of assessment phenomena, highlighting the complexity of assessment systems. Using Polarity Thinking™ might help us to sustainably manage these tensions.

## Polarity thinking™: application to assessment

As indicated in the previous section, increasing pressures for accountability and cost‐effectiveness drive tensions that arise from the complex interplay among competing or conflicting goals, beliefs and values, each of which represent equally valid alternative views of assessment reality. Tensions operate at and across different levels and stakeholders. For example, tensions between learning and performance – or related tensions of accountability and psychological safety – can exist at the level of the individual (learner, teacher) or the group (clinical workplace, clinical microsystem), as well as at the levels of programme management and the (education or health care) organisation. Attempts to manage key dilemmas through simple ‘either–or’ solutions or ‘if–then’ strategies are likely to have limited effectiveness (or might even fail), purely because simple ‘ifs’ may be hard to find in complex assessment systems. Rather, we would argue that the many chronic issues in assessment and assessment reform reflect multiple tensions between poles and polarities that are all necessary over time and should be managed to achieve sustainable and positive outcomes.

Use of the Polarity Thinking™ model to manage polarities involves three essential steps – seeing, mapping and tapping/leveraging – and requires the engagement of all key stakeholders in the process.^28^, ^29^, ^30^ The first step (*seeing*) is to know and accept that there are polarities, and to identify them and understand how they work. Key polarities can be identified by reviewing the assessment literature (e.g. as presented in this paper) and by holding conversations with stakeholders about the challenges and dilemmas they face in day‐to‐day assessment practice. The next step (*mapping*) is to give a name to the different values or points of view (i.e. poles) underlying a dilemma and, through collaborative conversation, to identify the positive (upside) and negative (downside) outcomes associated with each of the poles. This will encourage stakeholders to explore the dilemma from multiple perspectives and as a whole. Step 3 (*tapping/leveraging*) involves engaging stakeholders in discussions about strategies or actions that need to be adopted to maximise the upsides of *both* poles while avoiding the downsides of each pole. At the same time, for each of the poles, early warning signs can be identified to indicate that one pole is being overly focused on to the neglect of the other. Polarity Thinking™ in assessment design or reform may thus facilitate buy‐in from stakeholders through agreement upon strategies that must be adopted to achieve overarching goals and that accept and take multiple perspectives into account.

**Figure 2:**
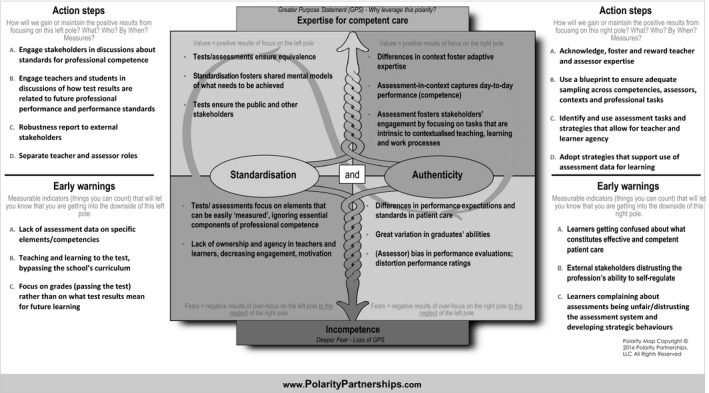
Example of a Polarity Map^®^ for standardisation and authenticity. In this example, the poles are standardisation and authenticity. Several upsides (top cells) and downsides (bottom cells) are described and action steps suggested to maximise the upsides (benefits). Early warning signs are listed related to the downsides (potential harms)

Management of polarities can be supported by the use of a Polarity Map^®^, which provides a structure for visualising and understanding polarities and how to address them. By definition, polarity maps are context‐specific – to some extent at least – as the map needs to represent organisational reality. Figure 2 presents a (fictitious) example of a Polarity Map^®^ for a common assessment polarity: the dilemma between standardisation and authenticity. Both these assessment approaches, although seemingly contradictory, are important in achieving the transformation of learners into high‐quality health care professionals who are able to provide safe, high‐quality patient care.

**Figure 3:**
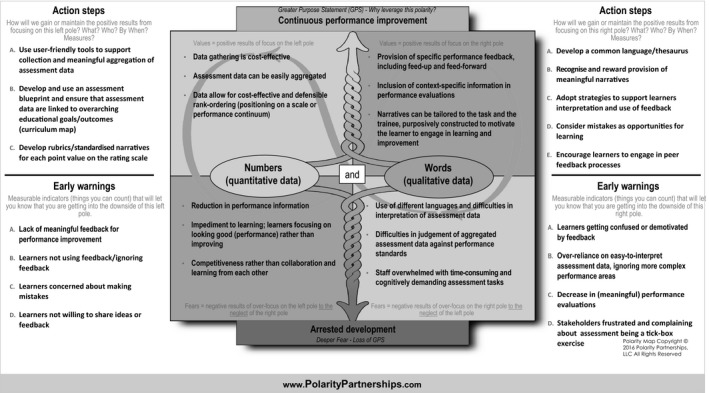
Example of a Polarity Map^®^ for numbers (quantitative data) and words (qualitative data). For each pole, the upsides (top cells) and downsides (bottom cells) are described with suggested action steps to maximise the upsides (benefits) and early warning signs related to the downsides (potential harms)

As the upper quadrants show, the poles of both standardisation and authenticity bring different, yet valuable, outcomes to the overarching purpose of ensuring health professionals are able to deliver high‐quality care, whereby standardisation ensures equivalence, clarity of goals and performance standards, and authenticity fosters adaptive expertise and the capturing of contextualised, day‐to‐day performance. The figure also shows the potential limitations of each pole (lower quadrants) and the loss of positive outcomes when one pole is focused on and the other neglected (diagonal quadrants). The action steps describe what education organisations and stakeholders need to do to keep both poles strong, whereas early warning signs represent the ‘symptoms’ that may emerge if there is too much focus on either authentic or standardised assessment. The map in Fig. 3 is filled in to provide an example of how the polarity between quantitative (numbers) and qualitative (words) assessment approaches can be managed.

Polarity maps are not necessarily comprehensive, but, rather, focus on the key, most impactful elements of each pole, including both the upsides (i.e. benefits) and downsides (i.e. potential harms). Much like the failure mode and effects analysis used in quality improvement initiatives,^86^, ^87^ polarity maps can be used proactively to anticipate the strengths and weaknesses of each pole when a training programme is planning to revise or implement an assessment, as well as to decide and agree upon strategies to maximise positive outcomes and monitor for early warning signs. By embracing both poles in assessment dilemmas, Polarity Thinking™ may thus provide a pathway out of the ‘either–or’ tension landscape in HPE and facilitate the evolving of assessment programmes to meet complex 21st century health and health care needs.

## Conclusions

Assessment polarities and tensions are likely to surface with the continued rise of complexity and change in education and health care organisations. With increasing pressures of accountability in times of stretched resources, assessment tensions and dilemmas will become more pronounced. Based on insights from organisation science and health care reform, we argue that we may need to add to our repertoire of strategies for managing key dilemmas in education and assessment design. The Polarity Thinking™ model may serve as a useful perspective for examining and addressing tensions in complex assessment systems. The use of this model encourages individuals to accept and leverage multiple views, rather than to engage in futile discussions about who or what is right. We do not want to pretend that all problems in assessment are to be viewed through the lens of Polarity Thinking™. This will not replace all ‘if–then’ solutions, but it may help to address the recurrent and persistent dilemmas that so very often compromise assessment practices. Its ‘both–and’ approach may help researchers to clarify the tensions that exist, how they fuel debates and assessment reform, and how actors navigate these tensions to achieve desirable outcomes. In programmatic assessment, for example, research questions may address tensions arising from the use of low‐stakes assessments for high‐stakes purposes, and how these impact on learner and assessor behaviours. Similarly, increasing use of narratives in competence assessment may raise questions about when, why and how to use grades or performance scores. Likewise, the adoption of Polarity Thinking™ and the reframing of problems in assessment from an ‘either–or’ to a ‘both–and’ perspective may help to advance our efforts to transform assessment systems to meet the needs of learners, education organisations and the public.

## Contributors

MJBG, CPMvdV and ESH worked collaboratively to develop the primary content of this paper. MJBG wrote the initial draft of the manuscript. All authors contributed to revisions of the initial draft for intellectual content and clarity. All authors approved the final manuscript for publication.

## Funding

none.

## Conflicts of interest

none.

## Ethical approval

not applicable.
